# Protective Effect of Aplysin Supplementation on Intestinal Permeability and Microbiota in Rats Treated with Ethanol and Iron

**DOI:** 10.3390/nu10060681

**Published:** 2018-05-27

**Authors:** Yan Ma, Ruiying Li, Ying Liu, Man Liu, Hui Liang

**Affiliations:** 1 Department of Human Nutrition, College of Public Health, Qingdao University, 38 Dengzhou Road, Qingdao 266021, China; mayanaa@163.com (Y.M.); liruiying1104@163.com (R.L.); lium_summer@126.com (M.L.); 2 Basic Medical College, Qingdao University of Medicine, 308 Ningxia Road, Qingdao 266071, China; shenghua005@163.com (Y.L.)

**Keywords:** ethanol, iron, aplysin, intestinal permeability, microbiota

## Abstract

Aplysin, a kind of phytochemicals or phytonutrients, is purified from red alga Laurencia tristicha. The present study aims to investigate the influence of aplysin on changes of intestinal permeability and microbiota induced by excessive ethanol and iron. Thirty male rats were randomly divided into three groups (10/group): control group (normal saline); ethanol + iron group as EI treated with ethanol (8–12 mL/kg/day) and iron (1000 mg/kg) in diet; EI supplemented with aplysin (150 mg/kg/day) group as AEI; the trial lasts for 12 weeks. The result showed that levels of plasma endotoxin, fatty acid-binding protein 2, D-lactic acid, diamine oxidase were increased in rats in the EI group; and significantly decreased by 14%, 17%, 26%, 16%, respectively (*p* < 0.05) in the AEI group after the 12-week aplysin treatment. Moreover, in the AEI group the amount of *Escherichia* coli and *Bacteroides fragilis* were higher, while the amount of *Lactobacillus*, *Bifidobacterium* and *Clostridium* were lower than those in the EI group. The expressions of iron transporters divalent-metal transporter 1(DMT1) and ferroportin 1(FPN1) were significantly upregulated in the EI group compared to those in the control group. In conclusion, aplysin could effectively improve intestinal permeability and intestinal flora disorder induced with excessive ethanol and iron.

## 1. Introduction

In the human body, iron is an essential element, but excessive iron intake can result in excessive production of oxygen free radicals [1], causing damage to different tissues and organs [2]. Chronic excessive alcohol intake causes liver injury, and over half of the patients with advanced alcoholic cirrhosis exhibited with high iron content in the liver [3], and a third of patients with chronic alcohol consumption also presented increased iron stores in the liver [4].

It has been found that different changes in intestinal microbiota and dysbiosis are caused by alcohol consumption in humans and animals [5,6]. Alcohol and its metabolites in the small intestine encounter a physical barrier, which plays a crucial role in preventing invasive pathogens into the body and protecting barrier integrity [7,8]. Alcohol intake may destroy intestinal barrier defense and lead to intestinal physical disruption [8,9]. It is well known that alcohol intake causes damage to intestinal barrier function, which can increase the mucosa permeability to many macromolecules [10,11]. Moreover, the close association of clinical expression of hemochromatosis and excessive alcohol consumption explained that the co-factor effect of iron and alcohol can lead to oxidative stress and hepatic injury [12]. However, it is unclear that co-treatment with ethanol and iron affects the intestinal permeability and gut microbiota.

**Figure 1:**
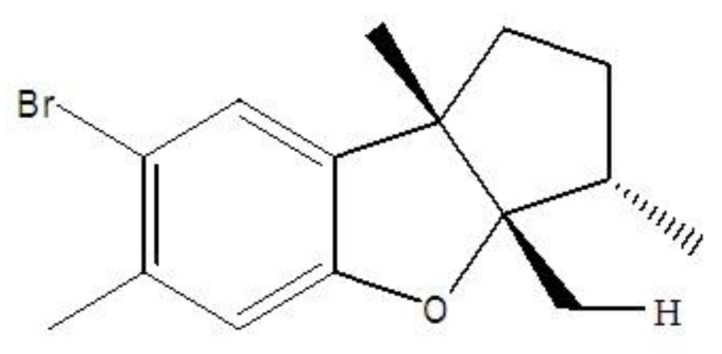
Chemical structure of aplysin.

Aplysin, a brominated sesquiterpene, is extracted from one kind of red alga named Laurencia tristicha (Figure 1). It has been previously described that its molecular formula is C15H19OBr and it has a molecular weight of 295 [13]. Aplysin potent biological activities include antitumor, anti-inflammatory, immunostimulation and antioxidant activities [14]. Previous study in our lab has demonstrated that a high dose aplysin treatment (150 mg kg^−1^ day^−1^) can significantly protect alcoholic liver injury [13] by repairing intestinal barrier function, normalizing fecal microbiota, and reducing inflammatory response and plasma endotoxin level in rats [15]. However, the potential effect of aplysin on the intestinal microflora and barrier function in rats exposed to iron and ethanol has not been explored.

Therefore, the present study aims to evaluate the effect of aplysin on the permeability of the intestine, the intestinal mucosal barrier, and intestinal microflora in rats exposed to iron and ethanol.

## 2. Materials and Methods

### 2.1. Animal and Ethics Statement

Animal Experiment Center (Qingdao, China) provided adult male Wistar rats weighting 180–220 g. The rats were kept on a 12 h light and 12 h dark at a relative humidity (50–60%) and a controlled temperature (22–25 °C). Standard rodent diet was fed to animals during the whole study. The animals were cared for according to international guidelines for the use and care of Laboratory Animals (Institute of Laboratory Animal Resources, Commission on Life Science, National Research Council, 1996). The animal use and care committee of Medical College of Qingdao University approved the animal study.

### 2.2. Experimental Designs and Animal Treatment

After a 1-week period of acclimatization, 30 rats were randomly divided into 3 groups (10/group): C, control group, fed with normal saline + normal diet; EI, ethanol and iron model group, fed with normal diet contained dietary high iron 1000 mg kg^−1^ and gavaged with ethanol 56% *v*/*v* (8 mL kg^−1^ day^−1^ 2 week + 12 mL kg^−1^ day^−1^ 10 week); AEI, Aplysin treatment group, normal diet contained dietary high iron 1000 mg kg^−1^ and gavaged with ethanol and aplysin (the dose of ethanol was as same as that in model group, aplysin 150 mg kg^−1^ day^−1^). The animals were supplemented with soya bean salad oil (1 mL) through gavage for 12 weeks in control group and model group. Rats were administrated by gavage with aplysin 150 mg kg^−1^ day^−1^ (soluble in soya bean salad oil) in the aplysin-treated group, following alcohol or saline lavage with 2 h interval for 12 weeks. Food consumption and body weight were monitored once a week and daily, respectively.

Aplysin is liposoluble and it can be dissolved in ethanol or other organic solvents. The Institute of Oceanology, Chinese Academy of Sciences identified the purity of aplysin—97.6%. Animal Experiment Center (Qingdao, China) provided the normal diet, which contained carbohydrates, protein, lipids, vitamins and other substances (Table 1). The type of alcohol (56% (*v*/*v*) ethanol) used in this study was Red Star Erguotou (Beijing Red Star Co., Ltd., Beijing, China).

After 12 weeks, the experiments used metabolic cages to collect the faeces of each rat after 12 h from the last intervention. Then the animals were sacrificed, samples of blood (or plasma), liver and small intestine tissues were taken and stored at −80 °C. Fresh tissues of the liver and small intestine were quickly excised, and the parts were fixed in 10% formaldehyde and embedded in paraffin.

### 2.3. Histopathological Analysis

The experiments processed formalin-fixed liver and small intestine tissues with hematoxylino-eosin (HE) staining, and examined morphological changes under light microscope (Olympus BX60, Japan). The ultrastructure of the intestinal tissue was observed by a JEM-1200EX transmission electron microscope (TEM) (JEOL, Tokyo, Japan).

### 2.4. Estimation of Serum Ferritin, Hepcidin and Iron-Related Protein Expression

The levels of serum ferritin (SF) and hepcidin (HEPC) were measured by a competitive enzyme immunoassay using ELISA kit, and procedures strictly followed the manufacturer’s instructions.

Subsequently, the protein expressions of divalent-metal transporter 1 (DMT1) and ferroportin 1 (FPN1) were performed by western blot analysis. Concisely, the experiment homogenized and lysed the intestine tissues to extract total proteins in radioimmunoprecipitation assay (RIPA) buffer including phenylmethanesulfonyl fluoride (PMSF). A BCA Protein Assay Kit was used to determine protein concentrations. An Enhanced chemiluminescence (ECL) plus system and a Molecular Imager ChemiDoc XRS System (Bio-Rad Laboratories, Hercules, CA, USA) were used to detect bound antibodies. Image Lab 2.0 Software (Bio-Rad Laboratories, Berkeley, CA, USA) identified and quantified optical densities of bands.

### 2.5. Measurement of Endotoxin Assay and Intestinal Mucosal Barrier Assay

CE TAL assay kit was obtained from Limulus Reagent Rlant Corp (Xiamen, China) and used to detect the plasma endotoxin levels. The experimental procedures were conducted following the manufacturer’s instructions. ELISA assay kits (Cloud-Clone Corp, Katy, USA) were used to determine D-lactic acid (D-LA), fatty acid-binding protein 2 (FABP2) levels and diamine oxidase (DAO) activity in plasma, in accordance with the instructions of manufacturer. The levels of DAO, D-LA, and FABP2 were identified from a standard curve. The data were reported as U L^−1^, μmol L^−1^ and ng mL^−1^, respectively.

### 2.6. Gut Microbiota Analysis

Genome DNA was extracted from 200 mg feces samples and stored at −20 °C. The contents of bacterial genome DNA were quantified by using real-time fluorescence quantification polymerase chain reaction (PCR) method, involving *Escherichia coil*, *Enterococcus*, *Bifidobacterium*, *Lactobacillus*, *Bacteroides fragilis* and *Clostridium tender*. The reaction system and reaction condition are similar to standard curve. According to the manufacturers’ instructions, the whole sequence was amplified for fluorescent real-time PCR with the kit (QIAgen, Shanghai, China). Primer sequences and annealing temperature of PCR amplification of specific bacterial are shown in Table 2.

### 2.7. Statistical Analysis

SPSS 18 (SPSS, Chicago, IL, USA) were used to analyze all data which were expressed as mean ± standard deviation (SD). One-way analysis of variance (ANOVA) was used to compare multiple groups with a Duncan’s multiple range test. It was considered to be statistically significant when results were at *p* < 0.05.

## 3. Results

### 3.1. Effects of Aplysin on Food Intake and Growth Performance

The information of the initial and final body weight (BW) was shown in Table 3. The initial body weight and diet intake among groups were no different at the end of 12 weeks (*p* > 0.05). However, the final body weight was decreased in rats treated with ethanol and iron compared with the control group, which was also attenuated by aplysin significantly (*p* < 0.05).

### 3.2. Effects of Aplysin on Serum Ferritin and Hepcidin

**Figure 2:**
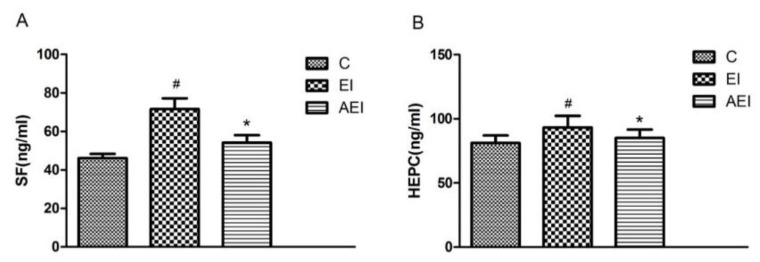
Effects of aplysin on serum ferritin (SF) and hepcidin (HEPC). (**A**) The serum ferritin in rats. (**B**) The serum hepcidin in rats. C: Control Group; EI: Ethanol and Iron Group; AEI: Aplysin (150 mg kg^−1^) with Ethanol and Iron Group. ^#^ *p* < 0.05 versus C group, * *p* < 0.05 versus EI group.

Serum ferritin and hepcidin of each group were shown in Figure 2. Chronic consumption of ethanol and iron (EI group) resulted in high levels of ferritin and hepcidin in serum (*p* < 0.05). Conversely, compared with the EI model group, the serum ferritin and hepcidin in the co-treatment of ethanol and iron rats with aplysin treatment were significantly lower (*p* < 0.05).

### 3.3. Effects of Aplysin on Intestinal Permeability

**Figure 3:**
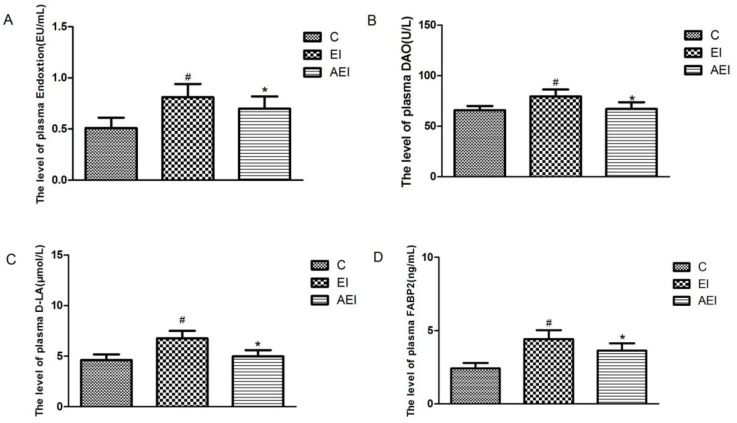
Effects of aplysin on intestinal permeability. (**A**) The plasma endotoxin level in rats. (**B**) Plasma diamine oxidase (DAO) level in rats. (**C**) Plasma D-lactic acid (D-LA ) level in rats. (**D**) Plasma fatty acid-binding protein 2 (FABP2) level in rats. C: Control Group; EI: Ethanol and Iron Group; AEI: Aplysin (150 mg kg^−1^) with Ethanol and Iron Group. ^#^ *p* < 0.05 versus C group, * *p* < 0.05 versus EI group.

As shown in Figure 3, compared with the control group, the level of endotoxin in plasma was significantly increased in the ethanol–iron-challenged rats (*p* < 0.05). Aplysin treatment daily to ethanol and iron-fed rats evidently decreased the endotoxin level (*p* < 0.05) (Figure 3A). Meanwhile, significantly higher DAO activity, and plasma levels of D-LA and FABP2 were observed in the ethanol and iron combined group in contrast with the control group (*p* < 0.05). In the aplysin treatment group, the plasma levels of DAO, D-LA, and FABP2 were reduced by 16%, 26%, 17%, respectively (*p* < 0.05), compared to those in co-dosed rats.

### 3.4. Effects of Aplysin on Intestinal Microbiota

**Figure 4:**
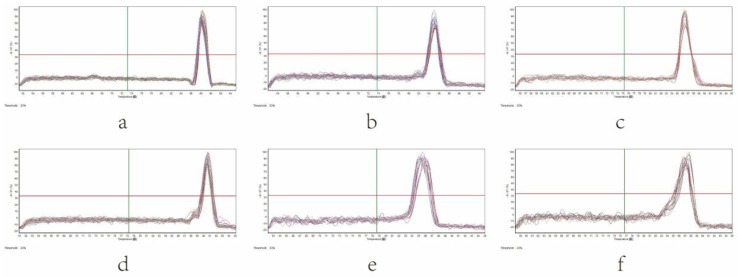
Melting curve of intestinal microbiota in different groups of rats by real-time q-PCR. (**a**) *Escherichia coil*, (**b**) *Enterococcus*, (**c**) *Lactobacillus*, (**d**) *Bifidobacterium*, (**e**) *Bacteroides fragilis*, (**f**) *Clostridium tender*.

**Figure 5:**
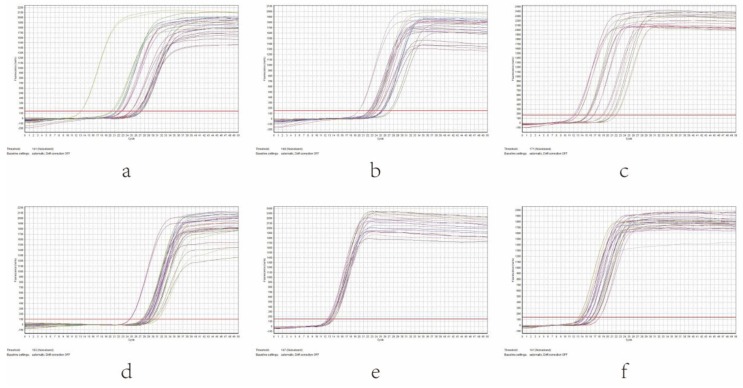
Amplification curve of intestinal microbiota in different groups of rats by real-time q-PCR. (**a**) *Escherichia coil*, (**b**) *Enterococcus*, (**c**) *Lactobacillus*, (**d**) *Bifidobacterium*, (**e**) *Bacteroides fragilis*, (**f**) *Clostridium tender*.

As shown in Figure 4, the results indicated that the amplification products were single after each quantitative PCR reaction. With the increase of cycle number, the fluorescence intensity was also enhanced. The curve tended to be parallel after a period of index amplification, namely a “platform effect” was observed, indicating that the corresponding relation between template copies at the index expansion period and the fluorescence cumulative value was the quantitative basis (Figure 5).

**Figure 6:**
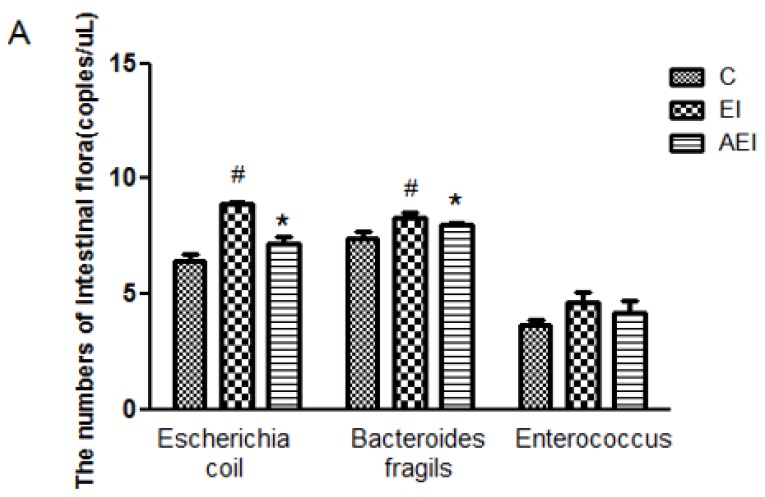

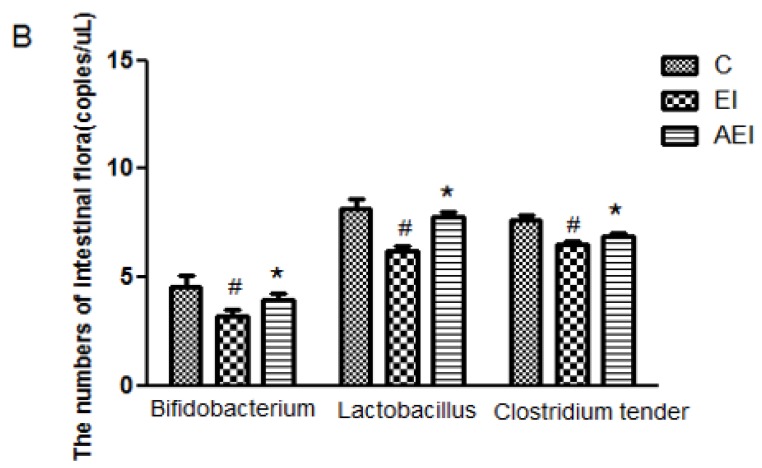
Effects of aplysin on intestinal microbiota. (**A**) The amount of *Escherichia coil*, *Bacteroides fragilis* and *Enterococcus* in feces of rats. (**B**) The amount of *Bifidobacterium*, *Lactobacillus* and *Clostridium tender* in feces of rats. C: Control Group; EI: Ethanol and Iron Group; AEI: Aplysin (150 mg kg^−1^) with Ethanol and Iron Group. ^#^ *p* < 0.05 versus C group, * *p* < 0.05 versus EI group.

The status of the intestinal integrity may also be reflected through fecal microbial culture. The amount of *Escherichia coil*, *Enterococcus*, *Bifidobacterium*, *Lactobacillus*, *Bacteroides fragilis* and *Clostridium tender* of different groups were given in Figure 6. In contrast with the control group, the amount of *Escherichia coil* and *Bacteroides fragilis* were elevated significantly (*p* < 0.05), but the amount of *Bifidobacterium*, *Lactobacillus* and *Clostridium tender* were all decreased significantly (*p* < 0.05) when co-treated by ethanol and iron. Aplysin effectively decreased the amount of *Escherichia coil* and *Bacteroides fragilis* in comparison with co-treatment of ethanol and iron (*p* < 0.05). Conversely, the amount of *Bifidobacterium*, *Lactobacillus* and *Clostridium tender* were obviously reversed by aplysin to ethanol and iron dosed rats. However, no statistical difference in the amount of *Enterococcus* among various groups was obtained (*p* > 0.05).

### 3.5. Effects of Aplysin on Pathological Changes of Liver and Intestine

**Figure 7:**
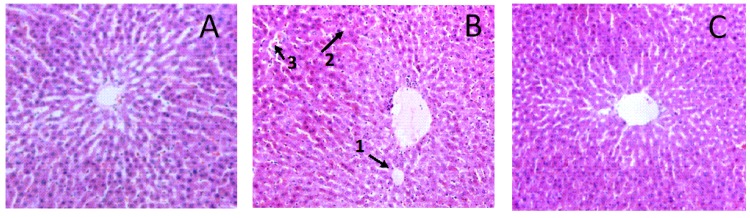
Ethanol-iron-induced liver histopathological changes in rats (Hematoxylino-eosin (HE) Staining, ×200). Normal architecture and structural intactness were shown in the control group. Hepatic cord derangement, inflammatory infiltration, microvesicular steatosis were shown in the EI model group. Aplysin (150 mg kg^−1^) treatment showed the architecture is almost normal. 1: Fat vacuoles; 2: Hepatocyte microvesicular steatosis; 3: Inflammatory infiltration. (**A**) Control Group; (**B**) Ethanol and Iron Group; (**C**) Aplysin (150 mg kg^−1^) with Ethanol and Iron Group.

According to HE staining for light microscopy of liver sections in different groups, the control groups showed normal lobular architecture. The hepatic cord was orderly. In addition, there was no steatosis in the liver (Figure 7A). Some extent of liver damage in the ethanol-iron treated group was indicated, such as the liver cells swelling, hepatic cord derangement, inflammatory infiltration, microvesicular steatosis and fat vacuoles (Figure 7B). However, in comparison with the co-treatment of ethanol and iron group, aplysin significantly improved these histopathological changes, and alleviated steatosis in the liver (Figure 7C).

**Figure 8:**
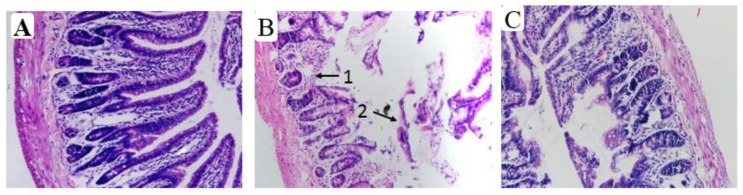
The pathological changes in the small intestine in rats (Hematoxylino-eosin (HE) Staining, ×200). It is indicated from HE staining that the mucosal tissue in the small intestine were normal and intact, and all intestinal villi were arranged neatly in rats of the control group. After ethanol-iron exposure, intestinal villus and glands were damaged significantly and many epithelial cells of small intestinal mucosa had fallen off. The pathological damages were improved after treated with aplysin. 1: Intestinal villus and glands were damaged significantly; 2: Large number of mucosal epithelial cell falls off. (**A**) Control Group; (**B**) Ethanol and Iron Group; (**C**) Aplysin (150 mg kg^−1^) with Ethanol and Iron Group.

According to HE staining for light microscopy of the intestine section in diverse groups, there was the intact and normal mucosal tissue in the small intestine, and intestinal villi arranged neatly in rats of the control group (Figure 8A). After ethanol-iron exposure, intestinal villus and glands were damaged significantly, mucosal epithelium and lamina propria separation, many mucosal epithelial cells fallen off, and intestinal villus hemorrhage and edema (Figure 8B). However, the intervention of aplysin improved the pathological injuries (Figure 8C).

**Figure 9:**
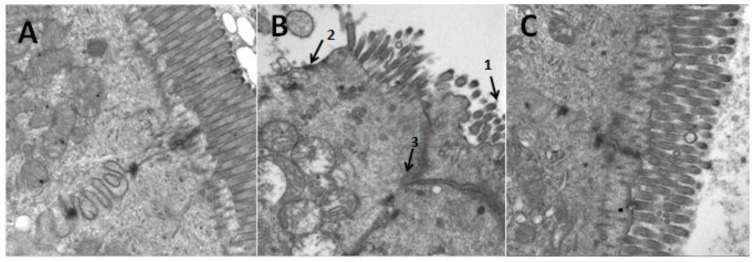
The ultrastructure of small intestine of rats (Magnification, ×20,000). In TEM, the tight junctions of the epithelial cells of intestine in the EI group were destroyed while the alterations of tight junctions in aplysin administrated rats were apparent. 1: Small intestine microvillis were sparse; 2: Small intestine microvillis fell away; 3: The cell connection structures were incomplete even disappear. (**A**) Control Group; (**B**) Ethanol and Iron Group; (**C**) Aplysin (150 mg kg^−1^) with Ethanol and Iron Group.

Further assessment of morphology with transmission electron microscopy (TEM) revealed that the small intestinal epithelial cells were arranged in neat rows. Rich, small intestinal villi and complete tight junctions between cells in the control group were clearly shown (Figure 9A). However, sparse small intestine microvilli and enlarged tight junctions were apparent by co-treatment of ethanol plus iron (Figure 9B). To varying degrees, the arrangement of small intestinal columnar epithelium and microvilli in the aplysin treatment group was improved and the swelling of the tight junctions was reduced (Figure 9C).

### 3.6. Effects of Aplysin on Expressions of Iron-Related Proteins in the Intestine

**Figure 10:**
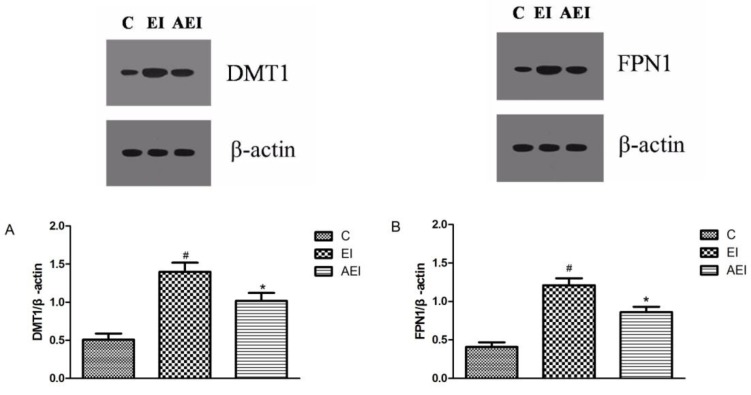
The protein expressions of divalent-metal transporter 1 (DMT1) and ferroportin 1 (FPN1). (**A**) The expression of divalent-metal transporter 1(DMT1); (**B**) The expression of divalent-metal transporter 1 (FPN1). C: Control Group; EI: Ethanol and Iron Group; AEI: Aplysin (150 mg kg^−1^) with Ethanol and Iron Group. ^#^ *p* < 0.05 versus C group, * *p* < 0.05 versus EI group.

The expressions of FPN1 and DMT1 proteins were up-regulated after co-treatment of ethanol plus iron compared to the control group (*p* < 0.05, Figure 10). Importantly, the expressions of FPN1 and DMT1 were clearly down regulated after aplysin treatment by 29% and 26% (*p* < 0.05).

## 4. Discussion

By a 12-week treatment with ethanol and iron in rats, our study demonstrated that supplementary aplysin significantly improved the histopathological damages and enhanced the intestinal mucosal barrier and maintained intestinal permeability. Our study found that an effective treatment may be associated with altered intestinal microflora. These results suggested that the development of intestinal damage was significantly prevented and the intestinal integrity was protected by aplysin.

We previously found that a continuous increase in the dose of alcohol (8–12 mL kg^−1^ day^−1^) caused damage in hepatic cells and it could overcome the tolerance induced by the same dose consumption of ethanol [13]. Ge et al. also reported that aplysin had the hepatoprotective effect on oxidative damage caused by ethanol, which could prevent alcoholic liver injury [13]. Xue et al. showed that aplysin could protect ethanol-induced hepatic injury, repair barrier function of the intestine and provide a low inflammatory response and a low level of plasma endotoxin [15]. Another study from a Chinese journal showed that the intestinal mucosal damage and lipid metabolism disorder caused by excessive iron exposure in rats were protected by aplysin [16]. Therefore, the current study further investigates the protective effects of aplysin on gut microbiota and intestinal permeability in rats co-treated with ethanol and iron.

The present study found that the final body weight was significantly lower in co-treatment of ethanol and iron in contrast with the control group, although each group had the same diet intake. It indicated that nutrition intake and absorption were affected, and the efficiency of calorie utilization was reduced in rats treated with ethanol and iron in a long-term period [17].

Intestinal health was investigated and discussed by measuring the barrier function of intestinal mucosa and the level of plasma endotoxin. Alcohol induced the damage to the barrier function of intestinal mucosa, increased intestinal permeability resulted from endotoxin and other adverse factors, and contributed to the endotoxemia in alcoholic liver damage [15,18]. In this study, the plasma endotoxin level was significantly higher in the co-treatment of ethanol plus iron group. However, ethanol and iron exposure with aplysin intervention showed that the plasma endotoxin level was lower than that in the model group (EI group). It suggested that aplysin may inhibit the high intestinal permeability induced by combined treatment of ethanol and iron.

The data in this study demonstrated that the D-LA, DAO and FABP2 levels in plasma were increased by long-term intake of ethanol and iron. DAO and D-LA are prevented from infiltratrating the portal blood, providing a barrier function by intact intestinal mucosa, so they are indices of high intestinal permeability [19]. Some studies documented that DAO activity and the D-LA level in the chronic ethanol intake were significantly increased [15,20]. Furthermore, decreasing the DAO activities and D-LA and FABP2 levels in plasma were observed after aplysin administration in the study. It showed that aplysin treatment could protect intestinal permeability and barrier function of intestinal mucosa in rats with ethanol and iron exposure.

Normal intestine permeability and the gut microbes are important factors for intestinal health. Fecal microbes are usually used to reflect gut microbes. The changes of intestinal microbiota and the proportion and number of bacteria in the small intestine were caused by alcohol intake, which was relative to ecological imbalance [5]. Moreover, intestinal dysbacteriosis resulted in deficiency of necessary micronutrients such as short chain fatty acids and others in the mucosa. It also led to the intestinal mucosal barrier being damaged and the intestinal permeability being increased [21]. It has been documented that the most frequent gut microbes in the intestinal tract are species of *Bacteroides fragilis*, Porphyromonas, *Bifidobacterium*, *Lactobacillus*, *Clostridium tender*, and *Escherichia coil* [22]. The results in this study showed a significantly higher amount of *Escherichia coil* and *Bacteroides fragilis* and a lower amount of *Bifidobacterium*, *Lactobacillus* and *Clostridium tender* when co-treated by ethanol and iron.

It has been suggested that Bacteroides can damage the intestinal health and may be carcinogenic [23]; it could use intestinal macrophages to stimulate the anti-inflammatory cytokine interleukin (IL)-10 production, as the most powerful inducer of regulatory T cells [24]. It has been reported that systemic and mucosal immune responses were modulated by certain *Bifidobacterium* and *Lactobacillus* species [25]. *Lactobacillus* has some positive physiological effects, which ferments carbohydrates to produce lactic acid. It can stop some harmful bacteria from the epithelial cells of the intestine and improve intestinal epithelial cells barrier function. It also can stimulate immunoglobulin production and strengthen the immune system of the host [26]. Our previous study showed that alcohol intake decreased the abundances of *Lactobacillus* [15]. Another study also suggested that the numbers of fecal *Bifidobacterium* were reduced, but *Escherichia coil* were increased in the ethanol group [27]. In a previous study, in rats of alcoholic steatohepatitis, it indicated that *Lactobacillus* GG treatment improved liver injury, gut leakiness and intestinal oxidative stress induced by alcohol [28]. Moreover, a study revealed that Fe deficiency increased the numbers of *Lactobacillus* with a trend towards decreased *Bacteroides* during in vitro colonic fermentation [29]. Zimmermann et al. investigated gut microbiota of African school children Fe supplemented for six months. It concluded that there were higher concentrations of *Enterobacteriaceae* and a lower amount of *Lactobacilli* in fecal samples of children with Fe supplemented biscuits which contained 20 mg Fe/d, compared to a control group with normal biscuits. A potentially more pathogenic gut microbiota profile which was produced by iron fortification is more related to increased gut inflammation [30]. In the present study, we also found that aplysin treatment produced a lower amount of *Escherichia coil* and *Bacteroides fragilis* and a higher amount of *Bifidobacterium*, *Lactobacillus* and *Clostridium tender* in feces and may have a tendency to restore the structure of intestinal flora in rats with combined treatment of ethanol and iron.

Serum ferritin and hepcidin, key regulators of iron metabolism, were detected in our study. The levels of serum hepcidin and ferritin significantly increased in co-treatment with ethanol and iron group compared with control. There was a similar result in which hepcidin protein and mRNA expression were higher in rats combined with ethanol and iron compared with the control group [31]. It suggested that excessive carbonyl iron caused primary iron overload, resulting in the high level of hepcidin expression. In contrast, chronic ethanol consumption decreased hepcidin expression, demonstrating that ethanol did not completely block the hepcidin expression induced by carbonyl iron [31]. Ioannou et al. suggested that chronic consumption of alcohol causes increased ferritin concentration and serum transferrin saturation, and even elevated iron stores in the liver [32]. Moreover, patients with chronic alcoholic disease exhibited increased intestinal iron absorption [33]. It was documented that the levels of serum iron, serum ferritin and transferrin saturation were increased gradually with the increases of iron levels in the diet [34]. Both ethanol and iron separately resulted in lipid peroxidation and oxidative stress, and damaged liver cells by the cumulative effects of iron and ethanol, even exacerbating liver injury in patients with alcoholic liver disease [35]. The results from the present study showed that aplysin significantly decreased serum ferritin and hepcidin induced by combined treatment of ethanol and iron.

Under HE and TEM observation, aplysin intervention alleviated fat accumulation and inflammation in the liver and the intestinal epithelial cells destroyed, and improved the pathological injuries, as previously suggested [15]. In recent years our understanding about intestinal iron absorption has improved by the identification of DMT1 and FPN1 [36]. We, therefore, found that DMT1 and FPN1 in the intestine were significantly upregulated after 12 weeks feeding with ethanol and iron. Harrison-Findik has hypothesized that chronic alcohol metabolism can result in down-regulating hepcidin expression, increasing intestinal absorption, and iron overload [37]. It has been documented that alcohol-mediated hepcidin suppression in the liver leads to high expression of ferroportin 1 and DMT1 in the duodenum [38,39]. It is possible that elevated ferroportin 1 expression in enterocytes may induce iron depletion, leading to the up-regulation of DMT1 [38]. Another study found similar results in that alcohol consumption decreased the serum hepcidin levels and led to elevation of DMT1 at the mRNA level and iron transporter ferroportin at the levels of mRNA and protein in the duodenum of patients with alcoholic liver disease [40]. There is a study showing that duodenal DMT1 levels in iron overloaded group were lower compared with the iron adequate group [41]. Harrison-Findik investigated the influence of iron together with alcohol in the intestine [39]. They demonstrated that the ferroportin expression in duodenum in the co-treated with ethanol and iron group elevated and reached the levels between iron-exposed and ethanol-exposed rats. It also observed that the down-regulation of hepcidin mRNA expression mediated by alcohol resulted in elevated expression of duodenal ferroportin in the iron-overloaded group. It has been shown that hepcidin binds to and degrades ferroportin protein, inhibiting iron uptake and release [42]. Except for in the iron-overloaded group, alcohol was shown to suppress iron-induced hepcidin expression, leading to increasing iron transport, as concluded by Harrison-Findik [39]. To our knowledge, it is the first time that aplysin treatment has been reported to evidently suppress the up-regulation of intestinal DMT1 and FPN1 expressions induced by co-treatment of ethanol plus iron.

However, the mechanisms between maintenance of microbiota composition and the barrier of intestinal mucosa are complicated and diverse. The proportion of different bacterial species or the intestinal flora composition plays a critical role in many cases. We should accurately analyze the fecal microflora both in quantitative techniques and in flora species. Therefore, analysis of the bacterial composition through 16S ribosomal DNA high throughput sequencing will be needed in our future studies.

## 5. Conclusions

The present study suggested that chronic co-treatment of ethanol plus iron could cause a variety of changes in the intestinal flora. The intestinal permeability was increased, the intestinal mucosal barrier was damaged and fecal microbial composition was imbalanced, which then contributed to a high plasma endotoxin level in rats as well as intestinal damage. However, aplysin intervention under ethanol and iron exposure could normalize fecal microflora composition, intestinal permeability and intestinal barrier function, and alleviate the high plasma levels of endotoxin, which showed a protective effect on ethanol-iron induced damage. These new findings suggest the rational use of marine food resources and provide a scientific basis for improving human health.

## Figures and Tables

**Table 1:** Composition of the diets.

The Composition of the Diets	(g/kg)
Sucrose	500.0
Casein	200.0
Corn starch	150.0
Cellulose	50.0
Corn oil	50.0
Mineral mix, AIN-76 (170,915)	35.0
Vitamin mix, AIN-76A (40,077)	10.0
DL-Methionine	3.0
Choline bitartrate	2.0
Ethoxyquin, antioxidant	0.01

**Table 2:** Primer sequences and annealing temperature of PCR amplification of specific bacterial.

Bacterial Species	Primer Sequences (5′­3′)	Lengths (bp)	Annealing Temperature (°C)
*Escherichia coil*	F: 5′-GTTAATACCTTTGCTCATTGA-3′	340	51
	R: 5′-ACCAGGGTATCTTAATCCTGTT-3′		
*Enterococcus*	F: 5′-ACTCGTTGTACTTCCCATTGT-3′	144	52
	R:5′-CCCTTATTGTTAGTTGCCATCATT-3′		
*Bifidobacterium*	F: 5′-GGGTGGTAATGCCGGATG-3′	442	61
	R: 5′-TAAGCGATGGACTTTCACACC-3′		
*Lactobacillus*	F: 5′- AGCAGTAGGGAATCTTCCA-3′	341	55
	R: 5′-CACCGCTACACATGGAG-3′		
*Bacteroides fragilis*	F: 5′- CTGAACCAGCCAAGTAGCG-3′	230	62
	R:5′-CCGCAAACTTTCACAACTGACTTA-3′		
*Clostridium tender*	F: 5′-GCACAAGCAGTGGAGT-3′	246	58
	R: 5′-CTTCCTCCGTTTTGTCAA-3′		

**Table 3:** Effects of aplysin on food intake and body weight.

Group (*n* = 10)	Food Intake (g)	Initial Body Weight (g)	Final Body Weight (g)
C	35.93 + 1.76	233.40 + 12.35	398.00 + 12.75
EI	34.72 + 1.36	236.55 + 8.23	350.30 + 13.76 ^#^
AEI	35.50 + 1.80	238.00 + 12.14	379.10 + 14.91 ^#,^*

^#^*p* < 0.05 versus C group, * *p* < 0.05 versus EI group. C: Control Group; EI: Ethanol and Iron Group; AEI: Aplysin (150 mg kg^−1^) with Ethanol and Iron Group.
